# The role of Tetraspanins in digestive system tumor development: update and emerging evidence

**DOI:** 10.3389/fcell.2024.1343894

**Published:** 2024-02-08

**Authors:** Shijie Shao, Zhen Bu, Jinghua Xiang, Jiachen Liu, Rui Tan, Han Sun, Yuanwen Hu, Yimin Wang

**Affiliations:** ^1^ Articular Orthopaedics, The Third Affiliated Hospital of Soochow University, Changzhou, China; ^2^ Department of General Surgery, Xinyi People’s Hospital, Xinyi, China; ^3^ Department of Gastroenterology, Kunshan First People’s Hospital Affiliated to Jiangsu University, Kunshan, China

**Keywords:** Tetraspanins, digestive system tumors, tumor metastasis, drug resistance, therapeutic target

## Abstract

Digestive system malignancies, including cancers of the esophagus, pancreas, stomach, liver, and colorectum, are the leading causes of cancer-related deaths worldwide due to their high morbidity and poor prognosis. The lack of effective early diagnosis methods is a significant factor contributing to the poor prognosis for these malignancies. Tetraspanins (Tspans) are a superfamily of 4-transmembrane proteins (TM4SF), classified as low-molecular-weight glycoproteins, with 33 Tspan family members identified in humans to date. They interact with other membrane proteins or TM4SF members to form a functional platform on the cytoplasmic membrane called Tspan-enriched microdomain and serve multiple functions including cell adhesion, migration, propagation and signal transduction. In this review, we summarize the various roles of Tspans in the progression of digestive system tumors and the underlying molecular mechanisms in recent years. Generally, the expression of CD9, CD151, Tspan1, Tspan5, Tspan8, Tspan12, Tspan15, and Tspan31 are upregulated, facilitating the migration and invasion of digestive system cancer cells. Conversely, Tspan7, CD82, CD63, Tspan7, and Tspan9 are downregulated, suppressing digestive system tumor cell metastasis. Furthermore, the connection between Tspans and the metastasis of malignant bone tumors is reviewed. We also summarize the potential role of Tspans as novel immunotherapy targets and as an approach to overcome drug resistance. Finally, we discuss the potential clinical value and therapeutic targets of Tspans in the treatments of digestive system malignancies and provide some guidance for future research.

## 1 Introduction

**FIGURE 1:**
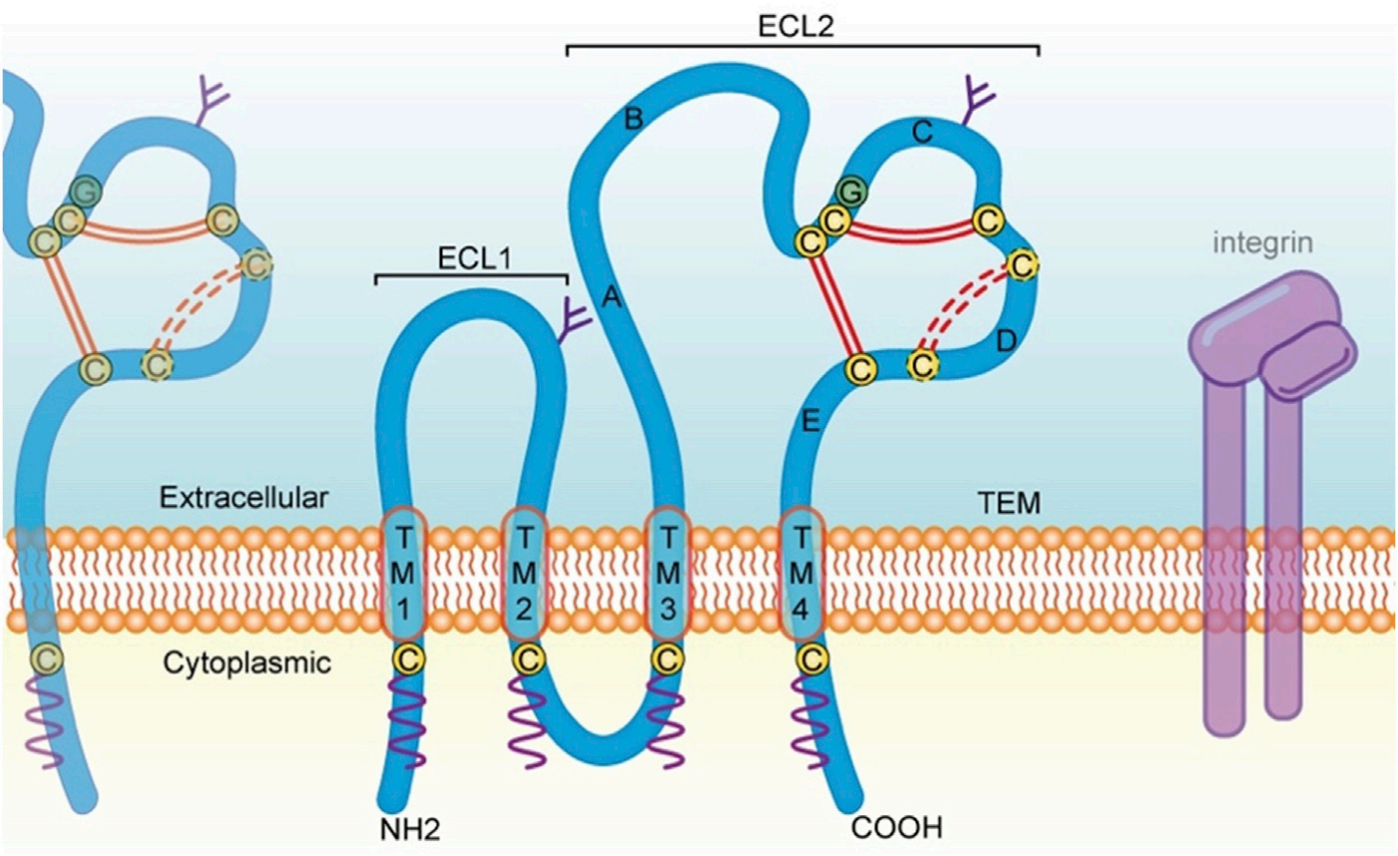
Structural feature of Tspans and discussed in the present review. Tspans contain four transmembrane domains (TM l-4). The ECL2 domain possesses five helices (helix A-E). Two antiparallel α-helices (A and E) in the continuity of TM3 and TM4 form the stalk of the domain. The A helix, B helix and E helix form a structure that is common to all Tspans. While the C and D helices are not structurally conserved and is therefore referred to as the variable domain of the ECL2, which contains several cysteine residues (yellow circles) including a CCG motif located after the B helix. Two disulfide bonds (double red line) are formed between these cysteine residues. There is one additional disulfide bond (dotted red line) in some Tspans, such as CD82 and CD151. Moreover, Tspans undergo palmitoylation (wavy purple line) at cysteine residues located near the intracellular border of the four transmembrane portions. On the cell membrane, Tspans interact with one another and other membrane proteins such as integrin, to form TEMs and serve a variety of functions including cell adhesion, invasion, motility, and signal transduction. Tspans, tetraspanin proteins; TM, transmembrane domain; ECL2, large extracellular loop. aa, amino acid; TEMs, tetraspanin-enriched microdomains.

In humans, 33 tetraspanin proteins (Tspans) belonging to the 4-transmembrane superfamily (TM4SF) have been identified so far (Susa et al., 2023). They are commonly found on the human white blood cell membrane, protruding 4–5 nm above the membrane, and they are often covered by the canopy of other glycoproteins due to their small size (Hemler, 2003). Individual Tspans are usually expressed at 30,000–100,000 copies per cell, and several different Tspans exist in nearly all cell and tissue types in humans (Hemler, 2003). Some Tspans such as CD151 and CD9, have an extensive, although not omnipresent, cell and tissue distribution, while others have a more restricted range of expression. For instance, Tspan7, Tspan9 and Tspan5 are enriched in brain. Tspan1, Tspan8 and Tspan11 are distributed in the intestine. CD37 and CD53 are found in lymphoid tissue, Tspan6 is present in the salivary gland, Tspan21 is located in the urinary bladder and prostate, while Tspan33 is abundant in the kidney (Deng et al., 2021). Structurally, Tspans are localized to the plasma membrane, where they cross four times (Toribio and Yanez-Mo, 2022). On the inner side of the plasma membrane, these proteins contain a small intracellular loop (composes of less than 30 amino acids) and a short cytoplasmic tail at both the N- and C-terminal, which contain palmitoylated cysteines (Florin and Lang, 2018). Some Tspans, such as CD151, contain a tyrosine-based sorting motif at the C-terminal that is crucial for subcellular localization and cell migration (Berditchevski and Odintsova, 2007; Liu et al., 2007). The outer side of the plasma membrane includes a highly conserved small extracellular loop (ECL1) and a large extracellular loop (ECL2) (Figure 1). Several Tspans, including CD9, CD81, and CD151, can interact with the classical RGD-binding site of integrin αvβ3 through the ECL2 domain (Yu et al., 2017).

Most Tspan proteins interact with partners, such as integrins, lipid kinases, tyrosine kinases, and PDZ-domain-containing proteins, to jointly establish the functional units called Tspan web or, more commonly, Tspan-enriched microdomains (TEMs) (Lekishvili et al., 2008; Termini and Gillette, 2017). Also, Tspans exist dimeric form, such as CD9^−^CD9 and CD151-CD81, which are the basic unit of TEM (Kitadokoro et al., 2002; Kovalenko et al., 2004). Different Tspans in TEM seem to have a specific feature in terms of dynamics, which may be due to the transient interaction of Tspans with gangliosides, resulting in different Brownian diffusion coefficient of Tspan proteins (Fernandez et al., 2021). Importantly, the TEM network is highly concentrated and serves crutial roles in modulating various cellular functions, including cell adhesion, motility, invasion, propagation, and signal transduction. Functionally, Tspans are extensively involved in human physiological and pathological processes, including the regulation of platelet aggregation, cell fusion, virus infection, immune response, allergic reaction, and especially cancer development. In terms of tumor biology, Tspans are essential at all the stages of tumor progression, exhibiting both pro-cancer and anti-cancer functions.

Digestive system tumors are an important part of global tumor morbidity and mortality, mainly including esophageal cancer, stomach cancer, liver cancer, pancreatic cancer, colorectal cancer, etc. With the improvement of people’s quality of life, the proportion of high calorie and low fiber in their diet is increasing, and the incidence of malignant digestive system tumors is rising year by year. Although there are more and more clinical treatment methods, the prognosis of patients remains poor (Siegel et al., 2020;et al., 2019). Therefore, the search for effective therapeutic targets is crucial for patients with digestive system tumor. As confirmed by an increasing number of studies, numerous Tspan proteins show abnormal expression in various malignant tumors of the digestive tract and play a vital biological role in the occurrence and development of the disease, including regulating tumor carcinogenesis, invasion, metastasis, proliferation, apoptosis and chemoresistance. Here, we review the current evidences on the function of Tspan family proteins in digestive system malignancies development and progression to provide some guidance for clinical treatment and future research.

## 2 Tspans in metastasis of digestive system tumors

### 2.1 CD82

CD82 (also known as Tspan27 and KAI1) was initially identified in 1995 by Dong et al in human prostate cancer. It is abundantly expressed in the human prostate, liver, and lung, but weakly expressed in the heart and gastrointestinal tract (Dong et al., 1995). CD82 is involved in various biological processes and diseases, including bone marrow homing and leukemia cell survival (Marjon et al., 2016; Ji et al., 2017; Saito-Reis et al., 2018; Ji et al., 2019), platelet aggregation (Uchtmann et al., 2015), viral infection (Florin and Lang, 2018), tuberculosis (Koh et al., 2018), rheumatoid arthritis (Bernard, 2018; Neumann et al., 2018) and muscular dystrophy (Alexander et al., 2016). Additionally, Hall A et al found that CD82 expression is necessary for muscle stem cell activation and supports dystrophic muscle function (Hall et al., 2020).

EMT is a reversible embryonic process that is aberrantly activated in tumor metastasis. Several studies have demonstrated that CD82 inhibits EMT and metastasis of digestive tumor cells through binding to extracellular matrix (ECM)-related molecules such as integrin and metalloprotease, and subsequently regulating their downstream signal transductions. In esophageal squamous cell carcinoma, CD82 was found to suppress malignant invasion and metastasis by decreasing the expression of TGF-β1, Smad2/3, MMP2 and MMP9, which in turn inhibit the degradation of ECM (Zeng et al., 2018). Similarly, a recent study confirmed that the anti-metastasis phenomenon in pancreatic cancer cells with CD82 overexpression may be due to the inhibition of EMT (Liu L. X. et al., 2019). In hepatocellular carcinoma (Dai et al., 2014), reported that anti-miR-197 inhibited HCC cell motility and invasion by directly targeting CD82. Consistently, downregulation of CD82 by miR-197 was found to increase gastric cancer cell aggressiveness and enhance EGFR phosphorylation, ERK1/2 phosphorylation and MMP7 expression (Xu et al., 2017). Also in gastric carcinoma, Zhang et al (Zhang et al., 2015) argued that the expression of CD82 was negatively correlated with miR-362-3p, and miR-362-3p promoted the motility and invasiveness of AGS and MKN45 gastric cancer cells via downregulation of the expression of CD82. In a further study, it was observed that TGF-β stimulated miR-362-3p and, in turn, decreased CD82 expression (Zhang P. F. et al., 2019). Hsih-Te et al (Yang et al., 2022) have demonstrated that CD82 was a new synthetic lethality target for KRAS mutations and demonstrated digitonin as a potential therapeutic agent through a bioassay database of KRAS mutant colon cancer cell lines. In addition, CD82 N-glycosylation at Asn157 inhibits EMT through inactivating the Wnt/β-catenin pathway and ultimately attenuates the ability of colorectal cancer cells to metastasize *in vitro* and *in vivo* (Zhu et al., 2022).

Notably, Luan et al (Luan et al., 2018) found that the highly conserved ECL1 domain of CD82 can inhibit the metastasis of multiple types of tumor cells. In a later study, He et al (He, Huang et al., 2020) demonstrated that this phenomenon may be attributable to the suppression of EMT, accompanied by downregulation of the Wnt/β-catenin pathway and upregulation of the Hippo/YAP signaling pathway. It was also suggested that ECL1 mimic peptide may be a promising candidate for developing anti-metastasis drugs (He et al., 2021). Intriguingly, the anti-metastatic capability of CD82 is possibly a result of its influences on cell mechanics and membrane tension, linked to the YAP/TAZ pathway and to caveolae mechanosensing (Ordas et al., 2021).

### 2.2 CD9

CD9 [Tspan29, MIC3, MRP-1, BTCC-1, and DRAP-27] is located on human chromosome 12p13.31. It is approximately 24 kD in length and is widely expressed on the plasma membrane, nucleus, endocytic compartment, and extracellular vesicles (EVs) (Andreu and Yanez-Mo, 2014). CD9 plays important roles in platelet activation and aggregation (Jennings et al., 1994), humoral immune response (van Spriel, 2011), fusion of ovum and sperm during mammalian fertilization (Le Naour et al., 2000; Miyado et al., 2000), allergic reaction (Koberle et al., 2012), and cell adhesion and migration (Powner et al., 2011). In recent years, considerable studies have shown that CD9 plays a pro-tumor and anti-tumor role in cancer development. In the following sections, we will focus on the latest advances of CD9 in tumor progression and metastasis, including various CD9-related binding proteins, downstream signaling pathways and transcriptional regulation.

A significant feature of CD9 is that it tends to interact with various transmembrane proteins such as integrins, EGFR, and other Tspans (e.g., CD151 and CD81) within TEMs (Berditchevski, 2001; Powner, Kopp et al., 2011). Therefore, the potential of CD9 to regulate cancer progression is attributed to its association with these molecules. Previous studies have indicated that CD9 and integrin β1 can play a vital role in the regulation of integrin-dependent adhesion strengthening and cell migration (Lazareth et al., 2019). A resent study confirmed that enhanced invasion and metastasis of colorectal cancer cells is the result of MMP-2 activation of the FAK^Tyr397^/ERK pathway via the CD9-α7β1 integrin complex (Kwon et al., 2021). Also, Santos MF et al (Guerra et al., 2022) have found that CD9 interacts with Trop-2 and enhances the growth of colorectal cancer cells through remodeling of β-actin cytoskeleton, proteolytic cleavage of E-cadherin, and activation of Raf-MEK-ERK and PI3K-PDK1-Akt pathways. Inhibition of CD9 expression in pancreatic ductal adenocarcinoma inhibited the uptake of annexin A6 extracellular vesicles into cancer cells and impaired annexin A6-induced cell migration and EMT processes via the p38 MAPK pathway (Nigri et al., 2022). Furthermore, CD9 could directly bind to ADAM10, ADAM9 and ADAM17 in pancreatic cancer cells. Antibodies targeting CD9 reduced cell surface trafficking of ADAMs and disrupted the interactions between CD9 and these ADAMs, impairing Notch activity and inhibiting the ability of cancer cells to migrate and grow (Lu et al., 2020). Paradoxically, recent studies have revealed CD9 itself exhibits an opposing role. Tang M et al (Tang et al., 2015) revealed that CD9 knockdown may play a pro-oncogenic role in pancreatic cancer-cell proliferation and metastasis, at least in part, via enhancing EGFR expression. Lysine specific demethylase 1 (LSD1) promotes the expression of CD9 through reducing intracellular miR-142-5p, which further leads to the inhibition of gastric cancer migration (Zhao, Fan, Li, Ren, Zhang, Liu et al., 2020). Moreover, CD9 knockdown can suppress activation of JNK signaling pathway, which facilitates hepatocellular carcinoma cell proliferation by downstream cyclin D1 and Bcl-2 factors (Li et al., 2020). These studies reveal the inhibitory role of CD9 in digestive system tumor growth and metastasis. The conflicting function in cancer progression may be attributed to the mechanism that involves its rather promiscuous binding and activation of certain CD9 partner molecules, allowing for different signaling pathways through those signaling transducers.

### 2.3 Tspan7

Tspan7 [CD231, TM4SF2, A15 and T-cell acute lymphoblastic leukemia (T-ALL)-associated antigen I], located on human chromosome Xp11.4, was first discovered by Takagi et al (Takagi et al., 1995) in 1995 as a highly specific marker for adult T-ALL. Tspan7 is extensively expressed in non-hematopoietic cells and strongly expressed in the brain, particularly the cerebral cortex and hippocampus (Hosokawa et al., 1999; Piluso et al., 2019). Stable expression of Tspan7 is crucial for the structure of hippocampal neurons and normal synaptic transmission (Bassani and Passafaro, 2012), and Tspan7 mutations are associated with intellectual disabilities, such as X-linked intellectual disability (Zemni et al., 2000; Bassani et al., 2012). Loss of Tspan7 in mice causes hippocampal abnormalities and cognitive impairment, which can be ameliorated by ampakine CX516 by promoting AMPA receptor activity (Murru et al., 2017). In addition, Tspan7 autoantibody can be used as a marker in type 1 diabetes and can distinguish individuals with latent autoimmune diabetes among adults characterized by the gradual decline in β-cell function, indicating its potential as a valuable target for immunotherapy in type 1 diabetes (McLaughlin et al., 2016; Walther et al., 2016; Shi et al., 2019).

Accumulating evidence suggests that Tspan7 plays a dual role as a tumor suppressor or pro-oncogenic factor in different types of cancer. For instance, Tspan7 has been shown to exert antitumor effects on bladder cancer by inhibiting tumor growth via the PTEN/PI3K/Akt pathway (Yu J. B. et al., 2020). Wuttig et al (Wuttig et al., 2009) reported that Tspan7 may be indicative of the disease-free interval and the number of pulmonary metastases in patients with clear-cell RCC (ccRCC), suggesting its association with tumor dormancy. Subsequent studies found that Tspan7 was positively associated with disease-free survival and tumor-specific survival in ccRCC patients, and that the expression of Tspan7 protein in vessels may serve as a potential prognostic marker in ccRCC (Wuttig et al., 2012). These findings suggest that Tspan7 inhibits tumor growth and metastasis and can be used as a prognostic marker. However, overexpressed Tspan7 in non-small cell lung cancer (NSCLC) cells was shown to markedly increase tumor volume *in vivo*, and significantly promote the migration of NSCLC cells by facilitating the EMT process (Wang X. et al., 2018). This paradoxical phenomenon also exists in digestive system tumors (Qi et al., 2020a). found that overexpression of Tspan7 attenuates the metastasis and growth of liver cancer LM3 cells. Meanwhile, a recent study indicated that high Tspan7 expression may be used to predict poor prognosis and high risk of metastasis in patients with pancreatic cancer, particularly those with Tumor-Node-Metastasis stages I and II (Luo et al., 2021). Further research will be necessary to explore whether Tspan7 plays conflicting roles in cancer regulation due to its different chaperones or downstream signaling pathways.

### 2.4 Tspan8

The Tspan8 gene (CO-029 and TM4SF3), with a total length of 1,182 kb, is located on human chromosome 12q21.1. Tspan8, specifically expressed within the junction region of the ventral pancreas where the two ventral buds fuse, is crucial for dorsal-ventral pancreatic bud fusion during embryonic pancreatic development (Jarikji et al., 2009). Tspan8 is also expressed in the stomach and duodenum, and it is required for acinar, gastric, and duodenal development (Jarikji et al., 2009). Enhanced Tspan8 expression identified in injured distal tubules of the kidney may contribute to tubule repair by facilitating migration and invasion of tubule cells (Hirukawa et al., 2014). Moreover, Tspan8 is expressed in the hippocampus and cerebellum of the brain, and participates in the development and maintenance of neuronal circuits through regulation of the expression of downstream genes, such as neurotrophic receptor tyrosine kinase 2 (Schartner et al., 2017). Tspan8 was also found to be positively associated with the risk of type 2 diabetes (Prabhanjan et al., 2016; Christodoulou et al., 2019).

In recent years, several studies have investigated the pro-tumor role of Tspan8 in digestive system tumors. Tspan8 has been identified as a biomarker for pancreatic cancer, and its co-localization with CD151-α6β4 has been associated with decreased adhesion and enhanced motility of pancreatic cancer cells (Gesierich et al., 2005; Wang et al., 2013). SOX9 positively regulates endogenous Tspan8 expression at the transcriptional level and also leads to loss of cell matrix adhesion and increased invasion in pancreatic cancer cells (Li et al., 2021). In gastric cancer, Tspan8 was found to be positively modulated by EGF in a dose- and time-dependent manner, and silencing Tspan8 diminished the effects of EGF on cell proliferation and invasion (Zhu et al., 2015). Wei et al (Wei et al., 2015) reported that Tspan8 markedly enhances the invasion and growth of gastric tumor cells by promoting the phosphorylation of MEK1/2 and ERK1/2. Furthermore, Tspan8 also plays a vital role in enhancing gastric cancer metastasis via activating the EGFR/Akt pathway (Zhang et al., 2023). In hepatocellular carcinoma, the scaffold protein astrocyte elevated gene-1 (AEG-1) was found to increase the transcription of Tspan8 via activation of MEK/ERK signaling. Silencing Tspan8 significantly abolished AEG-1-induced metastasis of cancer cells, and decreased the tumor volume in xenograft mice models, possibly by suppressing angiogenesis (Akiel et al., 2016). In colorectal cancer, Tspan8 augments the stemness of cancer cells via direct interaction with β-catenin and formation of a positive Tspan8/β-catenin expression regulatory loop (Zhan et al., 2019). Also in colorectal cancer, LSD1 increases the expression of Tspan8 by interacting with the Tspan8 promoter and removing H3K9me2 from the promoter. In addition, Tspan8 overexpression not only facilitates the proliferation of colorectal cancer SW480 and SW620 cells but also promotes metastasis by facilitating the EMT process in an LSD1-dependent manner (Zhang et al., 2020).

### 2.5 Tspan9

Tspan9 (NET-5 and PP1057) was first identified in 2000 as a new member of the TM4SF (Serru et al., 2000). This gene is located on human chromosome 12p13.33-p13.32. Tspan9 is approximately 27 kD in length and is widely expressed in the heart, kidney, and placenta. Notably, Tspan9 regulates platelet activation by facilitating the lateral diffusion of the collagen receptor glycoprotein VI (Protty et al., 2009; Haining et al., 2017). Furthermore, Tspan9 promotes virus transport and membrane fusion during early α-virus infection (such as Sindbis virus and Semliki Forest virus) through the regulation of the early endosome compartment, suggesting that Tspan9 may represent an effective antiviral therapeutic target (Ooi et al., 2013; Stiles and Kielian, 2016).

Tspan9 is one of the least characterized members of the Tspan family. Nevertheless, recent reports on Tspan9 have focused on its role in inhibiting the development and progression of digestive tumors, particularly gastric cancer (Feng et al., 2016). Tspan9 overexpression has been found to suppress the secretion of MMP9 and urokinase plasminogen activator (uPA) by inactivating the ERK1/2 signaling pathway, ultimately inhibiting the metastasis and proliferation of gastric cancer cells (Li et al., 2016). Additionally, Tspan9 overexpression has been shown to inhibit the motility and aggressiveness of cancer cells by inhibiting the EMT process and blocking the activation of the FAK/Ras/ERK pathway. Furthermore, the extracellular matrix glycoprotein elastin microfibril interfacer 1 (EMILIN1) may exert a synergistic effect with Tspan9 on the migration and invasion of gastric cancer cells (Qi et al., 2019). A recent study demonstrated that Tspan9 specifically interacts with PI3K regulatory subunit 3 (p55), and the phosphorylation of Tspan9 at Tyr153 is crucial for this binding. By binding to p55, Tspan9 suppresses the activation of PI3K/Akt/mTOR signaling and promotes cell autophagy in gastric cancer, thereby inhibiting the resistance of cancer cells to 5-fluorouracil (Qi et al., 2020b). Tspan9 also plays a role in other digestive tract tumors, such as colorectal cancer and liver cancer. A latest research identified extracellular vesicle membrane proteins in common among colorectal cancer cell lines and altered plasma extracellular vesicle protein profiles in colorectal cancer patients, suggesting plasma extracellular vesicle Tspan9 as a novel biomarker panel for detecting early-stage colorectal cancer (Dash et al., 2022). In HCC, Tan S et al (Tan et al., 2022) conformed that hsa-miR-9-5p-mediated Tspan9 downregulation was related to tumor immune infiltration and poor prognosis.

### 2.6 CD151

CD151 (also known as Tspan24, PETA-3 and GP27) was initially discovered as a platelet surface antigen (Ashman et al., 1991). This gene is located on human chromosome 11p15.5, with a total length of 1,443 kb. Notably, the YRSL sequence located in the C-terminal cytoplasmic tail of CD151 is essential for CD151-induced angiogenesis and cell migration (Liu et al., 2007; Peng et al., 2013). CD151 is broadly expressed in several tissues, particularly in the endothelium, epithelium, muscle, and platelets (Sincock, Mayrhofer, and Ashman, 1997). In humans, CD151 is crucial for the integrity of the basement membrane in the skin and kidney (Karamatic Crew et al., 2004). The interaction of CD151 with multiple surface integrins, including α4β1 and αLβ2, promotes the migration of T cells and induces inflammatory bowel disease (Zelman-Toister et al., 2016). Additionally, CD151 is required for Ca^2+^ mobilization and contraction of airway smooth muscle (ASM) cells and may facilitate airway hyper-responsiveness and ASM contraction via promotion of G protein-coupled receptor-induced calcium and protein kinase C signaling (Qiao et al., 2017).

Numerous recent studies have demonstrated that CD151 plays a carcinogenic role in digestive system tumors. In mice, high expression of CD151/MMP9 is associated with enhanced neoangiogenesis and an increased number of pulmonary metastatic lesions, while in patients with HCC, it has been linked to a higher recurrence rate (Shi et al., 2010). Kim JH et al (Kim et al., 2016) found that CD151 is a target of miR-199a-3p and that an increase in CD151 expression caused by reduced miR-199a-3p may contribute to promoting HCC cell migration and invasion. CD151 was also found to be upregulated by Mortalin, and silencing Mortalin impaired the TEMs of CD151 and suppressed the metastasis of CD151-overexpressed HCC cells (Liu X. et al., 2019). Consistently, small nucleolar RNA host gene 3 (SNHG3) overexpression enhance the EMT and sorafenib resistance of HCC cells through promoting CD151 expression (Zhang, Wang, Wu, Wu, Huang, Liu et al., 2019). Furthermore, PIK3C2A 3’untranslated region acts as a trans-activator to stimulate CD151 expression by competing miR-124 binding. The miRNA response elements in PIK3C2A 3’untranslated region can regulate CD151 through the competing endogenous RNA mechanism, thereby promoting the metastasis and growth of HCC cells (Liu et al., 2016). In colorectal cancer, CD151 has been reported to promote the migration and invasion of HT29 and HCT116 cells through a crosstalk involving Wnt signaling, LGR5 and CEACAM6 via inactivating TGF-β1 (Yang et al., 2021).

### 2.7 Other Tspans in digestive system tumor

Tspan1, a novel member of the TM4SF, is abundantly expressed in many types of cancers, including liver, gastric, colon, esophageal, and especially pancreatic cancer. It has also been related to promoting the occurrence and metastasis of digestive system tumors. In recent studies, Tspan1 has been demonstrated to promote the metastasis and growth of pancreatic cancer cells by positive regulation the FAM110A/HIST1H2BK/G9a axis (Huang et al., 2022). Similarly, Tspan1 contributes to pancreatic cancer cell migration and invasion through promoting MMP2 expression via PLCγ (Zhang Q. et al., 2019). Also in pancreatic cancer, Tspan1 was found to be negatively regulated by miR-573 and miR-216a, and upregulated Tspan1 contributed to the metastasis and proliferation of cancer cells (Wang et al., 2020; Wang et al., 2021). Consistently, Tspan1 was demonstrated to promote the EMT process and metastasis of cholangiocarcinoma via interacting with integrin α6β1 and activating the PI3K/Akt/GSK-3β/Snail/PTEN pathway (Wang Y. et al., 2018). Furthermore, Tspan1 was found to be negatively regulated by miR-573, and upregulated Tspan1 contributed to the invasion and proliferation of gastric cancer cells (Lu et al., 2015).

In gastric cancer, Tspan31 was found to be upregulated in tumor tissues, and silencing Tspan31 effectively inhibited migration and proliferation of GC cells by impairing the METTL1/CCT2 pathway (Ma et al., 2022). Also, Takashima Y et al (Takashima et al., 2022) demonstrated that Tspan31 knockdown inhibits cell metastasis of gastric cancer cells via inactivating the Akt/Snail pathway, leading to the suppression of EMT and metastasis. In hepatocellular carcinoma, Tspan5 has been demonstrated to facilitate tumor metastasis and the EMT process via activating Notch signalling (Xie et al., 2021). Consistently, knockdown of Tspan31 significantly suppresses HCC cell migration and invasion via inhibiting the activation of the Akt/p-GSK3β/β-catenin pathway (Wang et al., 2017). Conversely, CD63 was found to be downregulated in HCC tissues and be related to clinicopathological parameters of HCC patients. CD63 overexpression may suppresses HCC cell migration and growth by disrupting the activity of the IL-6/IL-27/STAT3 axis (Yu et al., 2021). In colorectal cancer, Tspan12 was found to be upregulated compared to that in paracarcinoma tissues, and silencing Tspan12 obviously inhibited cell metastasis and growth, induced cell apoptosis of cancer cells, which could provide a novel promising therapeutic strategy against human CRC (Liu et al., 2017). Moreover, functional studies have demonstrated that Tspan15 binds to Beta-transducin repeat containing E3 ubiquitin protein ligase (BTRC) to trigger NF-κB nuclear translocation, which subsequently activates the transcription of several transfer-related genes, including MMP9, uPA, TNF-α, VCAM1, ICAM1, and CCL2, ultimately promoting oesophageal squamous cell carcinoma metastasis (Zhang et al., 2018).

**FIGURE 2:**
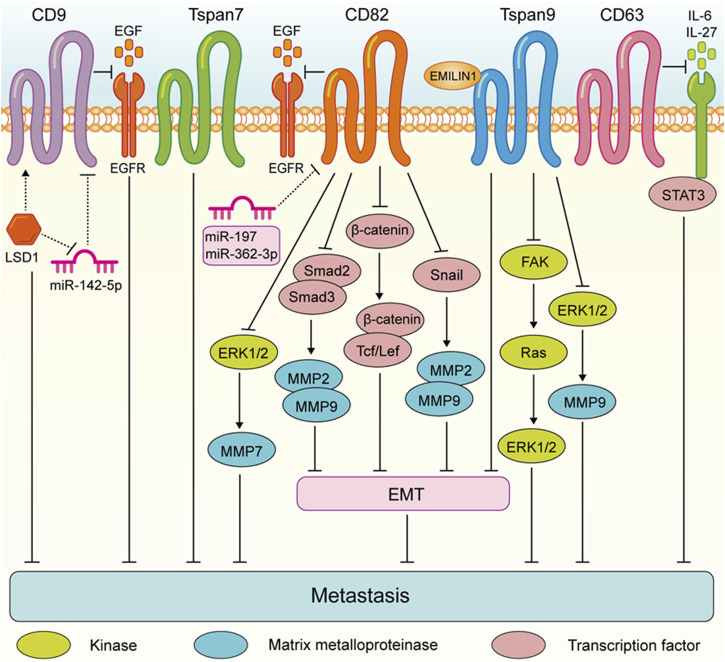
Tspans that suppress digestive system tumor cell metastasis. Especially, LSD1 promotes CD9 expression by reducing miR-142-5p, which further led to the inhibition of gastric cancer migration. CD82 suppresses gastric cancer cell aggressiveness by inhibiting the EGFR-ERK1/2-MMP7 expression. CD82 inhibits the metastasis of esophageal squamous cell carcinoma via suppressing the TGF-β1-Smad2/3-MMP2/9 pathway. CD82 N-glycosylation at Asn157 inhibits EMT through down-regulating the Wnt/β-catenin pathway and ultimately attenuates the ability of colorectal cancer cells to metastasize. CD82 inhibits the Snail-MMP2/9 pathway to repress EMT process invasion of pancreatic cancer cells. Tspan9 inhibits the aggressiveness of gastric cancer cells by inhibiting the EMT process and blocking the activation of the FAK-Ras-ERK pathway, and EMILIN1 exerted an anti-tumor effect by increasing Tspan9 expression. Tspan9 suppresses the metastasis of gastric cancer cells by inactivating the ERK1/2-MMP pathway. CD63 suppresses liver cancer cell migration by disrupting the activity of IL-6/IL-27/STAT3 axis.

**FIGURE 3:**
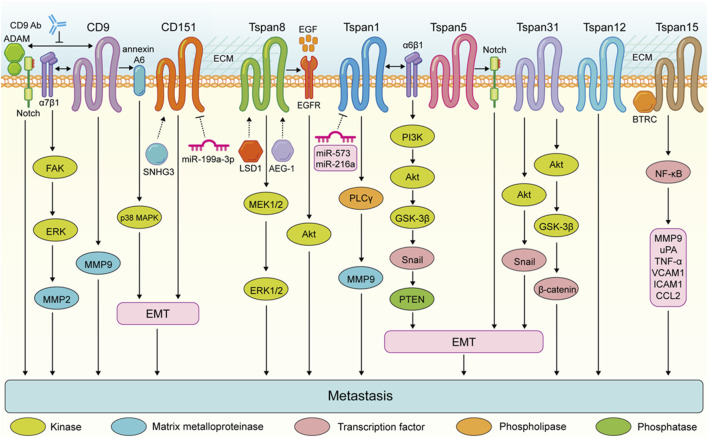
Tspans that promote digestive system tumor cell metastasis. Enhanced metastasis of colorectal cancer cells is the result of the FAK-ERK-MMP2 activation via CD9-α7β1 integrin complex. CD9 expression in pancreatic ductal adenocarcinoma promotes annexin A6-induced cell migration and EMT processes via the p38 MAPK pathway. The CD9-antibody targeting extracellular domain of CD9 disrupts the interactions between CD9 and ADAM family proteins and impairs Notch activity, and inhibits the ability of pancreatic cancer cells to migrate. Tspan8 enhances the invasion of gastric cancer cells by promoting the MEK1/2-ERK1/2 pathway. Tspan8 contribute to gastric tumor metastasis via activating the EGFR-Akt pathway. Tspan1 promoyes pancreatic cancer cell migration and invasion by promoting PLCγ-MMP9 pathway. Tspan1 facilitates the EMT process and metastasis of cholangiocarcinoma via interacting with α6β1 integrin and activating the PI3K-Akt-GSK3β-Snail-PTEN pathway. Tspan31 promotes the EMT and metastasis of gastric cancer cells via activating the Akt-Snail pathway. Tspan31 enhances liver cancer cell migration and invasion by inhibiting the activation of Akt-GSK3β-β-catenin pathway. Tspan15 binds to BTRC to trigger NF-κB nuclear translocation, which subsequently activates the transcription of several transfer-related genes, including MMP9, uPA, TNF-α, VCAM1, ICAM1, and CCL2.

These findings highlight the complex role of Tspan proteins in the metastasis of digestive system tumors. Generally, the expression of Tspan7, CD82, Tspan9, and CD63 are downregulated suppress the migration and invasion of digestive system tumor cells (Figure 2). Conversely, CD9, CD151, Tspan8, Tspan1, Tspan5, Tspan31, Tspan12, and Tspan15 are upregulated and enhance digestive system tumor cell metastasis (Figure 3). Consequently, further investigation is warranted to elucidate the underlying mechanisms of Tspans in various cancer types, with the aim of developing targeted drugs for clinical applications.

## 3 Tspans in chemoresistance

Resistance to therapy in cancer patients represents the primary barrier to achieving more effective treatments that can enhance both quality of life and survival. One of the major factors contributing to tumor relapse is resistance to chemotherapy. Previous studies have shown that ectopic expression of CD9 in small cell lung cancer (SCLC) cells represses integrin β1-dependen cell motility (Funakoshi et al., 2003) and facilitates apoptotic cell death via the inactivation of PI3K/Akt signaling (Saito et al., 2006). Furthermore, CD9 expression is upregulated and cell motility is reduced when SCLC cells are exposed to cisplatin or etoposide, and monoclonal antibodies targeting CD9 trigger apoptosis of chemicalresistant cells (Kohmo et al., 2010). These findings suggest that the absence of CD9 contributes to the highly malignant phenotype and increased susceptibility to apoptosis in SCLC.

Notably, the development of cancer and its subsequent acquisition of chemotherapeutic resistance are significantly influenced by cell-cell interactions and paracrine signaling between the tumor and its surrounding microenvironment, which includes endothelial cells, fibroblasts, and mesenchymal stem cells (MSCs) (Ridge et al., 2017). A previous study demonstrated that the activation of adipose-derived MSCs enhances their CD9 expression, thereby increasing their regenerative potential and proliferation (Kim et al., 2007). Furthermore, through *in vivo* co-cultured of bone marrow-derived MSCs (BMMSCs) and triple-negative breast cancer cell HCC 1806, Ullah et al generated tumors containing a new hybrid cell (DP-HCC1806:BMMSCs) that exhibited chemoresistance to doxorubicin and 5-fluorouracil. They showed that CD9 plays a crucial role in the interaction between BMMSCs and HCC1806 within the tumor microenvironment by regulating the expression of tumor resistance-related proteins such as CXCL12, CCL5, CCR5, and BCRP, ultimately leading to an enhancement of drug resistance in DP-HCC1806:BMMSCs (Ullah et al., 2019). Overall, CD9 facilitates the development of drug resistance in cancer cells and inhibits apoptosis of chemoresistance cells. In addition, CD9 also promotes drug resistance by mediating tumor microenvironments, such as the interaction between MSCs and tumor cells. Hence, targeting CD9 with monoclonal antibodies may offer insights into a new therapeutic approach to improve the prognosis of cancer patients undergoing chemotherapy.

Tspan8 has been shown to facilitate β-catenin expression and its translocation into the nucleus through interaction with Notch2, thereby enhancing multidrug resistance and reducing apoptosis in the gastric carcinoma SGC-7901/DDP cell line by activating the Wnt/β-catenin pathway (Li et al., 2018). Beyond digestive tumors, Pan et al demonstrated that Tspan8 knockdown suppressed the proliferation and migration of glioma cells by inhibiting the activation of FAK, and increased sensitivity to temozolomide (Pan et al., 2015). In breast carcinoma, Tspan8 overexpression promoted the stemness and drug resistance of breast cancer cells by facilitating the activation of the Sonic Hedgehog signaling pathway (Zhu et al., 2019). Moreover, in NSCLC, decreased Tspan8 expression resulted in G_2_/M-phase arrest and induced apoptosis of H1299 cells by decreasing the expression of cell cycle checkpoint kinase (CDK2/4) and cyclin D1, and increasing the expression of the pro-apoptotic proteins Bax and PARP (Dong et al., 2016).

For other TM4SF members, it was found that the glycosylation of CD63 was found downregulated by the knockdown of RPN2 (which is part of an N-oligosaccharyle transferase complex) in breast cancer, and silencing CD63 suppressed the chemoresistance and aggressiveness of tumor cells (Tominaga et al., 2014). In small cell lung cancer, Tspan12 has been reported to promote chemoresistance under the regulation of miR-495 (Ye et al., 2017). Floren M et al (Floren et al., 2020) demonstrate that CD82 overexpression contribute to chemoresistance through increasing p38 MAPK activation downstream of PKCα and β1 integrin mediated signaling, leading to acute myeloid leukemia cell survival. Downregulation of CD81 expression by aza/pano sensitizes acute lymphoblastic leukemia cells to chemotherapy and disrupts bruton tyrosine kinase (BTK) phosphorylation (Quagliano et al., 2020). Furthermore, in cisplatin-resistant head and neck squamous cell carcinoma, Tspan1 knockdown inhibited cell chemoresistance, EMT process, autophagy, proliferation and Src activation, while induced apoptosis *in vitro*. *In vivo*, Tspan1 inhibition decreased the metastatic spreading of cisplatin-resistant tumors and reduced tumorigenesis (Garcia-Mayea et al., 2020). Overall, targeting Tspans represents a promising strategy for suppressing chemoresistance in cancer therapy.

## 4 Tspans in cancer immunology

Immunotherapy has transformed and revitalized cancer treatment. Immune cells from both the innate and adaptive immune systems infiltrate the tumor microenvironment (TME) and regulate cancer progression. T cells are the focal point of tumor immunology research due to their potent tumor-killing abilities.

Antigen presentation is essential for T cell immune monitoring of cancer cells. MHC II molecules (MHC-II) drive CD4+T cell activation, while peptide presentation of endogenous expression proteins on MHC Class I molecules (MHC-I) drives CD8+T cell activation. Specialized antigen-presenting cells (APCs), such as B cells, monocytes, and dendritic cells (DCs), internalize and process antigens to produce immunogenic peptides capable of presenting antigens to T lymphocytes. Immunogenic peptid-binding MHC complexes (pMHC) on APCs bind to T cell receptors (TCR) to activate antigen-specific T cells (Rock et al., 2014).

Certain Tspans play vital roles in antigen presentation and cell migration in immune responses, including CD9, CD82, CD81, CD37, CD53, CD63, CD151, Tspan5, Tspan8, etc. CD9 has been reported to be necessary for the association of heterologous MHC II, a specialization that would facilitate the formation of MHC II multimers expected to enhance T cell receptor stimulation by DCs (Unternaehrer et al., 2007). Knockout of CD9 in mice enhances TNF-α production and macrophage infiltration in the lung after intranasal administration of lipopolysaccharide (Suzuki et al., 2009). CD9 induces MHC-II retention on cell surface via reducing MHC-II recycling and promoting MHC-II trafficking (Shi et al., 000). Moreover, CD82 expression increases after monocyte-derived DCs and bone marrow-derived DCs (BMDCs) activation, supporting MHC-II stable interactions and maturation between T cells and spleen DCs or BMDCs by suppressing the activation of RhoA (Jones et al., 2016). In a study by Gartlan KH, knocking out CD37 significantly inhibits DCs migration, which is a possible cellular mechanism for poor immunity in CD37^−/−^ mice (Gartlan et al., 2013). Additionally, CD53 ligation promote the adhesion of NK cells and reduce degranulation of NK cells in response to tumor target cells (Todros-Dawda et al., 2014).

In contrast, some Tspans such as CD63 and CD151, have shown inhibitory effects on T cell activation. Knockdown of CD63 in B lymphocytes continuously activates CD4+T cells by enhancing exosome production (Petersen et al., 2011). DCs lacking CD151 expression were overly irritating to T cells (Sheng et al., 2009). Moreover, CD82, CD53 and CD81 have been found to bind to MHC-II molecules in B-cell lymphoma cells, but their roles need to be further studied (Yuan et al., 2022). However, there have been few studies on the relationship between Tspan and MHC I complexes. One latest reserch confirmed that Tspan5-mediated MHC-I clustering was required for optimal CD8+T cell stimulation. Tspan5-MHC I clusters facilitate T cell activation may via their effects on TCR and CD8 clustering (Colbert et al., 2022).

Despite a large body of evidence that Tspans serve as a vital regulator of immune cells, there is a lack of direct evidence on the function of Tspans in tumor immunology. One recent study reported that Tspan8 can be used as a specific target candidate for chimeric antigen receptor T cells (CAR-T) against pancreatic cancer. Tspan8-specific CAR-T cells significantly reduced tumorigenesis in subcutaneous xenotransplantation models. The potential mechanism of Tspan proteins in anti-tumor immunotherapy needs to be further explored (Schafer et al., 2021).

## 5 Tspans in cancer therapy

As more detailed information about the relationship between Tspans and cancer becomes available, we hope that the TM4SF can be a promising target that provides an approach for clinical cancer therapy. It has been established that monoclonal antibodies (mAbs) specific to Tspans have great clinical value. To date, the prevailing view is that mAb against Tspans can inhibit cancer progression. Several murine or human antibodies have been generated to explore the therapeutic role of Tspan in different types of cancer. For example, anti-Tspan8 mAb (Ts29.2) suppressed colorectal tumor growth in a xenograft model and did not induce direct toxicity (Ailane et al., 2014). Even more notable is the fact that TS29.2 radiolabeled with lutetium-177 or indium-111 was also found to suppress colorectal tumor growth *in vivo* (Maisonial-Besset et al., 2017). Besides, a mAb targeting the LEL of Tspan8 inhibited the incidence of ovarian metastases *in vivo* and diminished tumor invasion *in vitro* (Kim et al., 2015; Park et al., 2016). For other TM4SF member such as CD9, several mAbs including GR2110, VJI/10 and VJI/20 suppressed the trans-endothelial migration of melanoma cells (Longo et al., 2001). In a xenograft model, the anti-CD9 antibody (ABL6) also inhibited the growth of human gastric cancer cells (Boucheix et al., 1983; Nakamoto et al., 2009). Notably, an anti-CD9 mAb (PAINS-13) that breaks the interaction of CD9 with integrin β1 reduced the growth of a human colon cncer xenograft more effectively than VJI/20 alone (Gutierrez-Lopez et al., 2003). These studies indicate that Tspan can be used as a potential therapeutic target for antibody therapy in Tspan-expressing cancer. However, research in clinical application is in its infancy, certain safety issues including individual suitability, side effects, variability of monoclonal antibodies and off-target effects, must be taken into consideration before the widespread application of targeted therapies. Furthermore, some mimics such as CD82 ECL1 mimic peptide (He, Ma, Wang, Luan, Li, Huang et al., 2021) and CD9-binding peptide (RSHRLRLH) (Suwatthanarak et al., 2021), may also provide an effective anti-metastasis approach to integrated cancer therapy.

By regulating multiple signaling pathways (Table 1), Tspans play crucial roles in cancer cell EMT, proliferation, migration, invasion, apoptosis, autophagy, neoangiogenesis and drug resistance. Blocking signal transduction is undoubtedly an effective means of treating cancer. It has recently been reported that inhibitors of multiple pathways with different targets and mechanisms of action (such as Wnt, Notch, Hedgehog, and Hippo pathways) are currently in different stages of clinical trials (Clara, Monge, Yang, and Takebe, 2020). Although some drugs targeting these signaling pathways have been approved for the market, the potential of these drugs in the treatment of solid tumors remains a research hotspot. In addition, the overexpression of some miRNAs contributes to the metastasis of digestive system tumor cell and poor prognosis via down-regulating Tspans’ transcription [for example, CD82 with miR-197 in hepatocellular carcinoma (Dai et al., 2014) and gastric cancer (Xu et al., 2017), CD82 with miR-362-3p in gastric cancer (Zhang et al., 2015), CD9 with miR-142-5p in gastric cancer (Zhao et al., 2020), Tspan9 with hsa-miR-9-5p in hepatocellular carcinoma (Tan et al., 2022)]. In contrast, some miRNAs restrain cancer progression through targeting Tspans [for example, CD151 with miR-199a-3p (Kim et al., 2016) and miR-124 (Liu et al., 2016) in hepatocellular carcinoma, Tspan1 with miR-573 and miR-216a in pancreatic cancer (Wang et al., 2020; Wang et al., 2021), with miR-194-5p in cholangiocarcinoma (Wang X. et al., 2018), and with miR-573 in gastric cancer (Lu et al., 2015)]. However, based on the miRDB database (https://mirdb.org/index.html), there are no regulatory correlations between miR-197 and CD82, miR-142-5p and CD9, as well as miR-573 or miR-216a and Tspan1(Liu and Wang, 2019; Chen and Wang, 2020). Therefore, the specificity of miR-197 on CD82, miR-142-5p on CD9, and miR-573 or miR-216a on Tspan1 needs to be re-examined. Overall, regulating these miRNAs might also be beneficial for the treatment of cancer. Tspans can also be used as therapeutic targets to increase drug sensitivity or to overcome drug resistance.

**TABLE 1:** Tspans and signaling molecules in cancer.

Tspans	Oncogene	Cancer type	Cell signaling	(Ref.)
CD82	−	Esophageal	TGF-β1/Smad2/3/MMP2/9	Zeng et al. (2018)
	−	Gastric	EGFR/ERK1/2/MMP7	Xu et al. (2017)
	−	Colorectal	Wnt/β-catenin	Zhu et al. (2022)
CD9-α7β1	+	Colorectal	FAK^Tyr397^/ERK/MMP2	Kwon et al. (2021)
CD9-Trop-2	+	Colorectal	PI3K-PDK1-Akt、Raf-MEK-ERK	Guerra et al. (2022)
CD9-ADAM	+	Pancreatic	Notch	Lu et al. (2020)
CD9	−	Liver	JNK/cyclin D1/Bcl-2	Li et al. (2020)
CD9	+	Lung	PI3K/Akt	Saito et al. (2006)
Tspan7	−	Bladder	PTEN/PI3K/Akt	Yu et al. (2020a)
	+	Osteosarcoma	FAK/Src/Ras/ERK1/2	Shao et al. (2022b)
Tspan8	+	Gastric	MEK1/2/ERK1/2	Wei et al. (2015)
	+	Gastric	EGFR/Akt	Zhang et al. (2023)
	+	Colorectal	β-catenin	Zhan et al. (2019)
	+	Glioma	FAK	Pan et al. (2015)
	+	Breast	Sonic Hedgehog	Zhu et al. (2019)
Tspan8-Notch2	+	Gastric	Wnt/β-catenin	Li et al. (2018)
Tspan9	−	Gastric	ERK1/2/MMP9	Li et al. (2016)
	−	Gastric	FAK/Ras/ERK1/2	Qi et al. (2019)
	−	Gastric	PI3K/Akt/mTOR	Qi et al. (2020a)
	+	Osteosarcoma	FAK/Ras/ERK1/2	Shao et al. (2022a)
CD151	+	Colorectal	TGF-β1	Yang et al. (2021)
	+	Osteosarcoma	GSK-3β/β-catenin/MMP9	Zhang et al. (2016)
Tspan1	+	Pancreatic	FAM110A/HIST1H2BK/G9a	Huang et al. (2022)
	+	Pancreatic	PLCγ/MMP2	Zhang et al. (2019c)
	+	Cholangiocarcinoma	PI3K/Akt/ GSK-3β/Snail/PTEN	Wang et al. (2018a)
Tspan31	+	Gastric	METTL1/CCT2	Ma et al. (2022)
	+	Gastric	Akt/Snail	Takashima et al. (2022)
	+	Liver	Akt/p-GSK3β/β-catenin	Wang et al. (2017)
Tspan5	+	Liver	Notch	Xie et al. (2021)
CD63	−	Liver	IL-6/IL-27/STAT3	Yu et al. (2021)
CD81	+	Osteosarcoma	Akt/ERK	Mizoshiri et al. (2019)
Tspan15	+	Osteosarcoma	PI3K/Akt	Yu et al. (2020b)

Oncogene, +; tumor suppressor.

Once digestive system tumors spread to the bone, they are not only difficult to cure, but also often accompanied by serious skeletal-related events, such as pain, an increased risk of fracture and hypercalcemia, which seriously reduce the quality of life and survival of patients. Since bone metastasis not only causes a huge burden on the life and psychology of patients, but also seriously affects the staging of tumors and the choice of treatment plan, early diagnosis and treatment of patients with bone metastasis of digestive tract tumors are of great value for their prognosis. With the rise of targeted therapy, a growing body of research suggests that Tspan proteins may be useful anti-metastatic targets for bone tumor. In a study by Mizoshiri, CD81 knockout osteosarcoma 143B cells showed significantly reduced tumor formation and lung metastasis in mice, accompanied by diminished Akt and ERK phosphorylation (Mizoshiri et al., 2019). Consistently, CD81-negative patients with plasma cell myeloma have a better prognosis. CD151 has been demonstrated to promote osteosarcoma pulmonary metastasis, and depletion of CD151 in cancer cells significantly suppressed integrin β1, FAK, p-mTOR, and p70s6 levels as well as Akt, p56 and p38 phosphorylation (Wang et al., 2016). Moreover, Zhang et al (Zhang, Wang et al., 2016) reported that knockdown of CD151 decreased MMP9 secretion by decreasing phosphorylated GSK-3β and β-catenin, in turn decreasing osteosarcoma cell invasiveness and motility. Consistently, Tspan1 was found to be negatively regulated by miR-491-3p, and downregulated Tspan1 attenuated the invasion and proliferation of osteosarcoma cells (Duan et al., 2017). Also, Tspan15 was found to be negatively regulated by miR-16-5p, and downregulated Tspan15 significantly decreased the metastasis and growth of osteosarcoma MG63 cells via inhibiting the PI3K/Akt pathway (Yu X. et al., 2020). Our latest research found that Tspan7 was able to interact with integrin β1 to augment FAK-Src-Ras-ERK1/2 pathway within osteosarcoma cells so as to drive enhanced cell metastasis (Shao et al., 2022a). Similarly, Tspan9 can interact with integrin β1 and facilitate osteosarcoma metastasis via the amplification of FAK/Ras/ERK1/2 pathway (Shao et al., 2022b). In general, Tspans may be early markers of bone metastasis in digestive system tumor, and targeting Tspan is a promising measure to inhibit the metastasis of malignant bone tumor in cancer therapy.

## 6 Conclusion

Further investigation into the structure and underlying molecular mechanisms of Tspans will enhance our understanding of the role of these membrane proteins in regulating cancer development. Tspan proteins interact with various molecules to form complex and massive TEMs that are involved in numerous essential cell activities. As noted above, given that Tspans play a bidirectional function in promoting digestive malignancy and bone tumor metastasis, blocking cancer-promoting Tspans or their downstream factors may present successful therapeutic targets in the futur.
